# Structural insights into pink-eyed dilution protein (Oca2)

**DOI:** 10.1042/BSR20230060

**Published:** 2023-07-25

**Authors:** Shahram Mesdaghi, David L. Murphy, Adam J. Simpkin, Daniel J. Rigden

**Affiliations:** 1 Institute of Systems, Molecular and Integrative Biology, University of Liverpool, Liverpool L69 7ZB, England; 2 Computational Biology Facility, MerseyBio, University of Liverpool, Crown Street, Liverpool L69 7ZB, U.K.

**Keywords:** albinism, AlphaFold 2, molecular modelling, protein structure

## Abstract

Recent innovations in computational structural biology have opened an opportunity to revise our current understanding of the structure and function of clinically important proteins. This study centres on human Oca2 which is located on mature melanosomal membranes. Mutations of Oca2 can result in a form of oculocutanous albinism, which is the most prevalent and visually identifiable form of albinism. Sequence analysis predicts Oca2 to be a member of the SLC13 transporter family, but it has not been classified into any existing SLC families. The modelling of Oca2 with AlphaFold2 and other advanced methods show that, like SLC13 members, it consists of a scaffold and transport domain and displays a pseudo inverted repeat topology that includes re-entrant loops. This finding contradicts the prevailing consensus view of its topology. In addition to the scaffold and transport domains, the presence of a cryptic GOLD domain is revealed that is likely responsible for its trafficking from the endoplasmic reticulum to the Golgi prior to localisation at the melanosomes. The GOLD domain harbours some known glycosylation sites. Analysis of the putative ligand binding site of the model shows the presence of highly conserved key asparagine residues that suggest Oca2 may be a Na^+^/dicarboxylate symporter. Known critical pathogenic mutations map to structural features present in the repeat regions that form the transport domain. Exploiting the AlphaFold2 multimeric modelling protocol in combination with conventional homology modelling allowed the building of plausible homodimers in both inward- and outward-facing conformations, supporting an elevator-type transport mechanism.

## Introduction

Albinism is a hereditary condition affecting the synthesis of melanin. The most prevalent and visually identifiable form of albinism is oculocutanous albinism. Oculocutanous albinism is a recessive disorder where affected individuals exhibit melanin deficiency in the skin, hair, and eyes. Oculocutanous albinism results from mutations in genes that code for proteins that are involved in melanin production. The gene affected is used to classify the type of oculocutanous albinism into one of the seven subtypes (oculocutanous albinism 1–7); oculocutanous albinism type 1:TYR, oculocutanous albinism type 2:OCA2, oculocutanous albinism type 3:TYRP1, oculocutanous albinism type 4:SLC45A2, oculocutanous albinism type 6:SLC24A5, oculocutanous albinism type 7:LRMDA and oculocutanous albinism type 5 gene is located on chromosome 4q24 [1]. Accurate diagnosis of the sub-type can only be achieved by a genetic screen [2]. The most prevalent form of oculocutanous albinism is oculocutanous albinism type 2 in which mutations in the OCA2 gene cause changes in the transmembrane protein p protein (Oca2) thereby impacting melanin production. Polymorphisms of the OCA2 gene have been shown to be major contributor to skin colour [3] and are thought to underlie blue eye colour in humans [4]. Oca2 is expressed in melanocytes and retinal pigment epithelium (RPE) where it is restricted to melanosomes.

Melanosomes are ‘lysosome-related organelles’ but are functionally and morphologically distinct from lysosomes as they have an acidic luminal pH [5] and possess cell-type-specific cargo proteins [6]. Trafficking pathways deliver these cargo proteins to immature melanosomes, a process which contributes to their maturation [7]. Oca2 is located in the mature melanosomal membrane where it has been shown to control chloride conductance across the lipid bilayer [8]. This chloride conductance is coupled to proton motive force, and is related to maintenance of the optimal luminal pH for the tyrosinase function involved in the production of melanin [8]. The currently accepted model, based on hydrophobicity profiles, describes Oca2 as a 12 transmembrane helix protein with two luminal loops and an N-terminal disordered cytoplasmic loop [9]. Oca2 is glycosylated in the N-terminal luminal loop and the N-terminal cytoplasmic loop of Oca2 possesses dileucine motifs; both of these features are important for the trafficking of Oca2 from the ER to the melanosomes through a series of intracellular compartments [10].

This study employs deep learning modelling methods to argue for a revised topology for Oca2. Deep learning methods such as DMPfold [11], trRosetta [12] and AlphaFold2 [13] build predicted protein structures by predicting protein features such as inter-residue distances and utilizing the predictions as restraints in the model building process. Benchmarking these methods has demonstrated that they work just as well for membrane proteins as they do for soluble proteins [11,14].

The modelling of Oca2 using AlphaFold2 predicts the presence of a pseudo inverted repeat that forms a pore region flanked by two highly conserved re-entrant loops. Additionally, a luminal loop preceding the first transmembrane helix is predicted to be a GOLD-like domain that allows trafficking through the Golgi from the endoplasmic reticulum to finally localise at the melanosomal membrane. The newly proposed topology shares features with sodium-carboxylate transporters (NaCT), which is supported by apparent sequence homology.

## Methods

### Hardware

Local ColabFold model building was performed on an Ubuntu 18.04.6 workstation AMD Ryzen Threadripper 2990WX 32 Core CPU (3.0 GHz) with 64GB RAM. GPU acceleration was performed by an ASUS TUF GeForce RTX 3080 OC LHR 12GB GDDR6X Ray-Tracing Graphics Card, 8960 Core, 1815MHz Boost.

### Pfam database screening

Searches using the sequence of Oca2 were made against the Pfam-A_v35.0 (RRID:SCR_004726) [15] database using the HHPred (RRID:SCR_010276) v3.0 server [16] with default parameters (-p 20 -Z 10000 -loc -z 1 -b 1 -B 10000 -ssm 2 -sc 1 -seq 1 -dbstrlen 10000 -norealign -maxres 32000 -contxt /cluster/toolkit/production/bioprogs/tools/hh-suite-build-new/data/context_data.crf) and eight iterations for Multiple Sequence Alignment (MSA) generation in the HHblits [16] stage.

### Structural database screening

Dali (Distance matrix alignment) (RRID:SCR_013433) v5.0 server [17] was used to screen the Protein Data Bank (PDB) [18] and the human proteins of the AlphaFold Protein Structure Database (AFDB) [19] for structural neighbours of Oca2. Pairwise alignments were also performed by the Dali server.

### Model building

An initial Oca2 model was obtained from the AFDB [19]. The construction of the inward-facing homodimeric form and attempted alternate conformations was performed with a local installation of ColabFold [20]. The outward-facing monomers were finally constructed by first building a homology model of Oca2 using an outward-facing structure of a homologue. The HHpred server was used to identify 6wtw as a close homologue (99.97% probability). The Modeller [21] functionality of the MPI bioinformatics toolkit server [16] was used to build the homology model. The homology model as a template along with custom MSAs of varying depths were used as inputs to a local installation of ColabFold. Five models at each MSA depth were constructed and the model with the highest mean pLDDT score was selected for examination. The outward-facing homodimer was constructed using a local installation of ColabFold with the Modeller homology model used as a template and an MSA with depth of 15 sequences.

### Multiple sequence alignments

MSAs were build using the HHblits server [16] using default settings. The reduction in MSA depth, as a strategy to assist exploration of conformational diversity in AlphaFold2 outputs [22], was achieved by randomly selecting sequences from the HHblits output.

### Docking

The Webina server [23] utilizing Autodock [24] was used to dock citrate into the putative binding pocket of Oca2. A docking box size of 35 × 35 × 35 Å was used with the default coordinates for the box centre. Prodigy [25] was used to perform docking rescoring. Electrostatics analysis was done with Adaptive Poisson-Boltzmann Solver (APBS) [26].

## Results and discussion

### Oca2 is a member of the IT Superfamily

Oca2 is an 838-residue transmembrane protein annotated in UniProt [27] with the Pfam domain CitMHS (PF03600). An HHpred search of the Pfam database reveals that Oca2 also possesses strong sequence similarity to other members of the Ion Transporter (IT) Superfamily with HHpred probability scores above 99.9% (Table 1).

**Table 1:** HHpred results for screen of Oca2 sequence against Pfam

Pfam Accession	Pfam Name	Probability	*E*-Value	Query HMM residue range	Template HMM residue range
PF02040.18	ArsB	100	1.90E-26	334-831	2–422
PF00939.22	Na_sulph_symp	100	3.50E-25	324-837	23–473
PF06450.15	NhaB	100	1.20E-24	328-831	42–503
PF16980.8	CitMHS_1	99.9	2.30E-23	332-829	11–442
PF07399.14	Na_H_antiport_2	99.9	1.50E-22	328-826	1–413
PF06808.15	DctM	99.9	6.00E-20	339-830	1–410
PF02447.19	GntP_permease	99.9	9.70E-19	329-834	1–443
PF03600.19	CitMHS	99.9	8.00E-19	342-773	1–348

ArsB protein is established as a bacterial arsenite efflux pump [28]. Although it is common for ArsB to complex with the ATPase ArsA to form an ATP-driven pump that expels arsenite [29] it has been shown that ArsB can function independently as an arsenite efflux pump by coupling with proton motive force [30]. Although Oca2 shows no sequence similarity with Cl^-^ transporters it has been demonstrated experimentally that Oca2 is required for melanosomal anion efflux contributing to the mediation of chloride-selective anion conductance which in turn modulates melanosome pH thereby regulating melanin synthesis [8].

### SLC13 members have a pseudo inverse repeat topology

The IT Superfamily is made up of both symporters and antiporters [31]. Experimental structures are available for some members, but no experimental structures are available for Oca2. To identify experimental structures of close evolutionary relatives to Oca2, the sequence was screened against the PDB using HHpred. There were three hits above 99.9% probability comprising members of Pfam families DASS (divalent anion sodium symporter) family sodium-coupled anion symporter, Solute carrier family 13 member 5 and NaDC (Table 2). Additionally, the AlphaFold2 model of Oca2 was screened against the full PDB using Dali. All hits above a *Z*-score of 35 were Na^+^ symporters in the inward conformation with the top hit being 7jsj, the Solute carrier family 13 member 5 and NadC (Supplementary Table S1). Furthermore, the AlphaFold2 model of Oca2 was screened against the AFDB of the human proteome using Dali; there were five hits above a *Z*-score of 30 for the conserved Pfam domain (PF16980); Solute Carrier Family 13 Members 1, 2, 3, 4, and 5 (Table 3). Thus, the results of HHpred sequence analysis and Dali structural similarity searches both suggest that Oca2 is a close evolutionary relative to members of Solute Carrier Family 13, sharing structural and potentially functional features.

**Table 2:** HHpred results for the screen of Oca2 sequence against the PDB

PDB Accession	Name	Probability	*E*-Value	Query HMM residue range	Template HMM residue range
6wtw_B	DASS family sodium-coupled anion symporter	100	5.00E-24	323-837	32–490
7jsk_B	Solute carrier family 13 member 5	99.99	8.20E-24	327-836	31–552
5ul9_D	Transporter, NadC family	99.9	3.70E-23	326-832	23–444

**Table 3:** Dali results for structural screen (residues 331-831) of Oca2 against Human AlphaFold database

Name	*Z*-Score
Human:Af-Q86YT5-F1 Solute Carrier Family 13 Member 5;	36
Human:Af-Q9UKG4-F1 Solute Carrier Family 13 Member 4;	34.6
Human:Af-Q8WWT9-F1 Solute Carrier Family 13 Member 3;	34.1
Human:Af-Q13183-F1 Solute Carrier Family 13 Member 2;	33.4
Human:Af-Q9BZW2-F1 Solute Carrier Family 13 Member 1;	29.7
Human:Af-O00337-F1 Sodium/Nucleoside Cotransporter 1;	10.6
Human:Af-Q9hAS3-F1 Solute Carrier Family 28 Member 3;	10.2
Human:Af-O43868-F1 Sodium/Nucleoside Cotransporter 2;	10.3
Human:Af-A6NH21-F1 Serine Incorporator 4;	9.1

**Figure 1:**
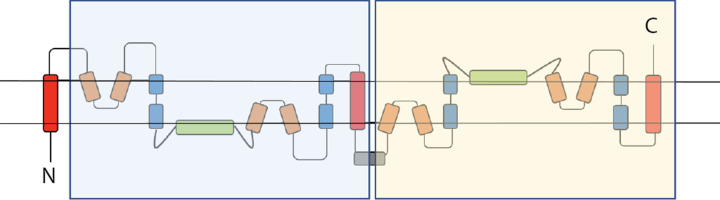
NaCT topology Shaded regions highlight the pseudo inverse repeat. Red: transmembrane helix; Orange: re-entrant loop; Blue: broken helix; Green: amphipathic helix; Grey: extra-membrane helix.

Solute carrier (SLC) proteins are integral membrane transport proteins that are classified into 66 families [32,33]. Members within each family have greater than 20% sequence identity. However, the homology between solute carrier families may be non-existent [34] as the basis for the introduction of a family as a solute carrier protein is related to functionality rather than an evolutionary link. Currently there is one structure available for a mammalian SLC13 protein; 562 residue long sodium-dependent citrate transporter (NaCT), SLC13 member 5. NaCT displays inverted repeat pseudo-symmetry relating the N-terminal half to the C-terminal half [35] with each repeat containing a re-entrant loop packing against a broken transmembrane helix, followed by a cytosolic amphipathic helix parallel to the membrane plane, a second re-entrant loop packing against a broken transmembrane helix then finally a transmembrane helix (Figure 1).

### Oca2 has a pseudo inverse repeat topology

**Figure 2:**
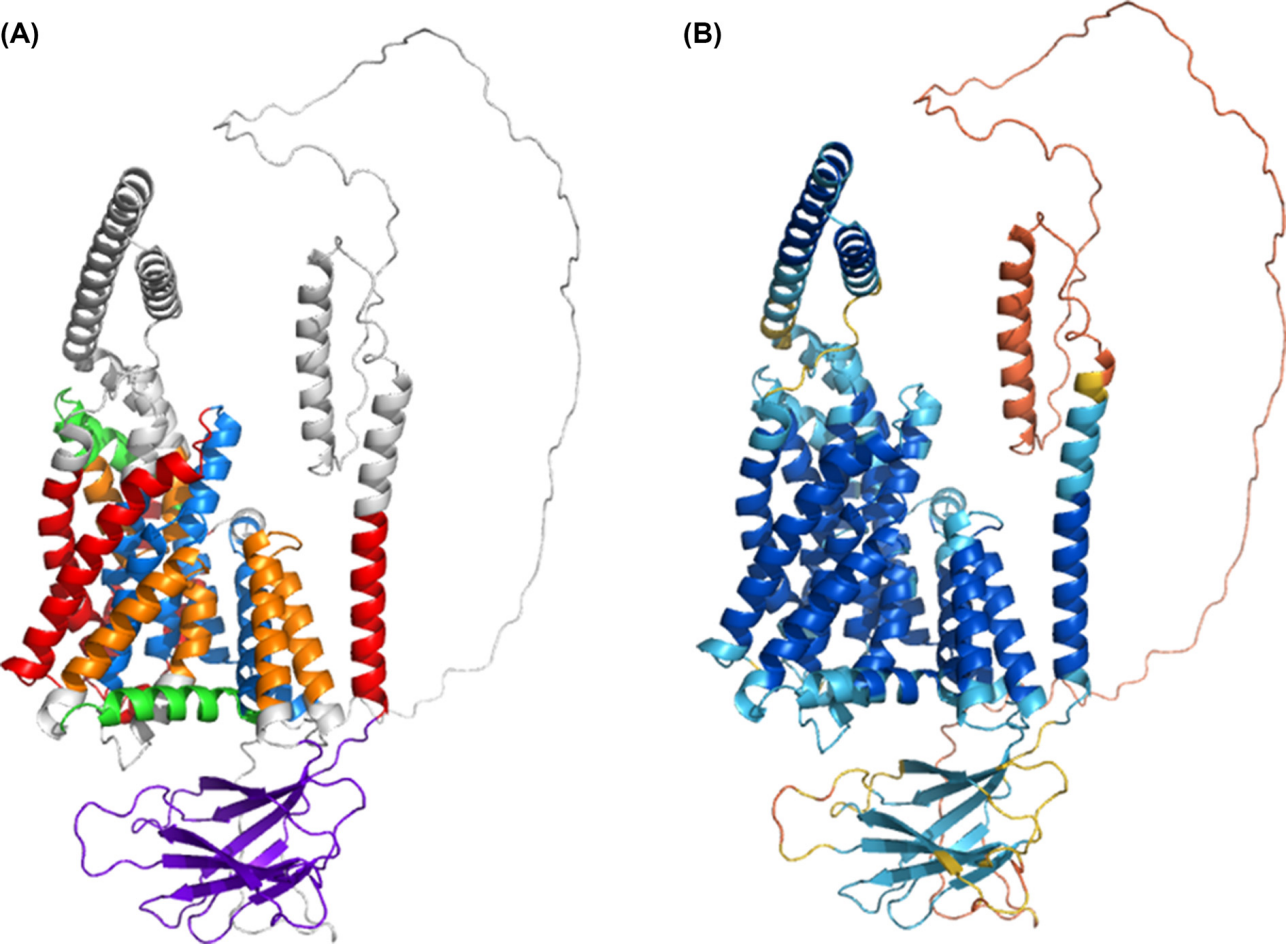
AlphaFold2 Oca2 model (**A**) Coloured by structural features - Red: transmembrane helix; Orange: re-entrant loop; Blue: broken helix; Green: amphipathic helix; Purple: ‘N-terminal luminal loop’ now annotated as a putative GOLD domain; Grey: extra-membrane helix. (**B**) Coloured by AlphaFold2 per-residue confidence score (pLDDT) between 0 and 100. pLDDT>90 (blue) to pLDDT<50 (red).

Examination of the Oca2 AlphaFold2 model (Figure 2) reveals a more complex topology compared with the currently accepted model of 12 transmembrane helices with two luminal loops and an N-terminal disordered cytoplasmic loop (Figure 4).

**Figure 3:**
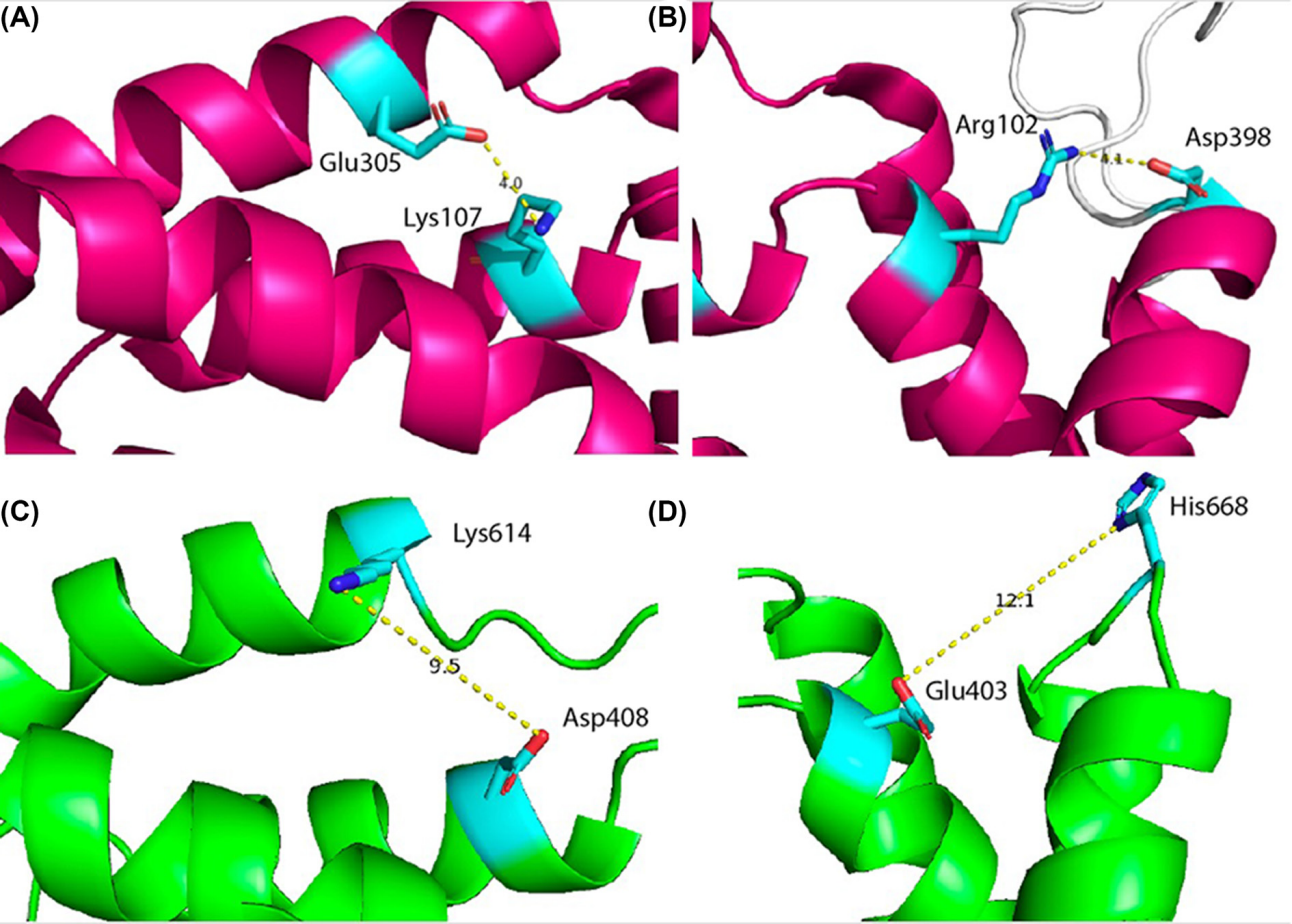
Stabilising salt bridges (**A**)Observed salt bridge stabilising the scaffold domain in NaCT (Lys107-Glu305). (**B**) Observed salt bridge stabilising the scaffold domain in NaCT (Arg102-Asp398). (**C**) Potential salt bridge in Oca2 (Asp408-Lys614). (**D**) Potential salt bridge in Oca2 (Glu403 with His668).

The AlphaFold2 model shares topological features with NaCT: a pseudo inverse repeat, each possessing a broken transmembrane helix, an amphipathic helix and a re-entrant loop packing against a broken transmembrane helix. Extrapolating functional annotations from homologues of Oca2 identified by the HHpred PDB screen indicates that each repeat unit of Oca2 possesses a transport domain made up of a re-entrant loop packed with transmembrane helix, where the transmembrane helix is broken in the centre. The amphipathic helices of each unit link the transport domain to a scaffold domain formed by the other helices of the conserved CitMHS domain. It has been shown in the experimentally characterised homologues that during the transport cycle, the two amphipathic helices are fixed in space with respect to the scaffold domain and cradle the transport domain during the conformational transition between the outward- and inward-facing states [31,36]. The rigidity of the Oca2 amphipathic helices could be achieved by salt bridges, between Glu403 with His668 as well as Asp408 with Lys614. Indeed, mutations of the equivalent residues (Arg102-Asp398 and Lys107-Glu305), in NaCT results in the abolition of substrate transport [35]. The distances between the potential salt bridge-forming residues in the Oca2 model are beyond the 4Å threshold distance for salt bridge formation. However, in both cases the residues are located adjacent to flexible disordered loops the movement of which may enable salt bridge formation (Figure 3).

**Figure 4:**
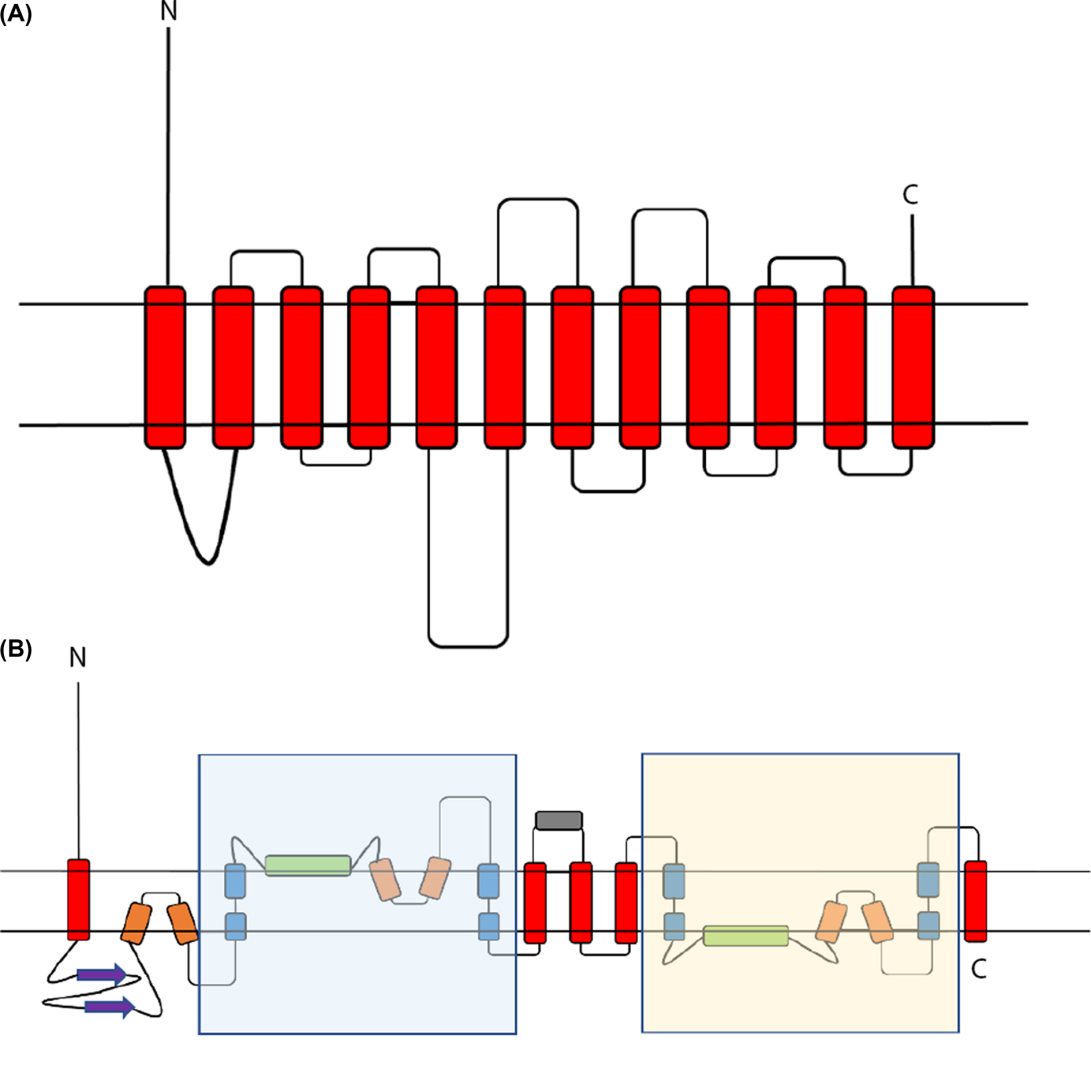
Oca2 topologies (**A**) Current consensus view of Oca2 topology [10]. (**B**) New proposed Oca2 Topology. Shaded regions are the pseudo inverse repeat. Red: transmembrane helix; Orange: re-entrant loop; Blue: broken helix; Green: amphipathic helix; Purple: ‘N-terminal luminal loop’ now annotated as a putative GOLD domain; Grey: extra-membrane helix. N marks the N-terminal and C marks the C-terminal.

Further inspection of the model identified additional structural features outside of the repeating units; a 171 residue long cytosolic N-terminal disordered region, a luminal 130 residue forming an eight stranded beta sandwich and a 95-residue cytosolic helical region separating the two inverse repeats (Figure 4).

### Oca2 dileucine motifs responsible for melanosome localisation are located on the disordered cytosolic N-terminal region

The model of Oca2 has an N-terminal disordered loop. AlphaFold2 models this loop with very low confidence (pLDDT < 50) and packs it against the transmembrane helices. As a result, when placed into a membrane bi-layer using the OMP server, the 170 residue long cytosolic N-terminal disordered region crosses the membrane which is not energetically favorable. Packing of intrinsically disordered regions against the main domain has been seen previously in models produced by AlphaFold2 [37].

An AlphaFold2 remodelling exercise was performed in order to attempt to obtain a model where the N-terminal disordered region does not transverse the membrane boundaries as defined by the OPM server [38]. The output provided 5 new models. The highest-ranking model with a pLDDT score of 72.9 showed the disordered loop mostly on the cytosolic side of the membrane with the exception of a 5-residue region [39–44]. This stretch crosses the OPM predicted membrane bi-layer boundary and therefore still represents an obvious error.

Previous experimental studies have identified this loop to be cytoplasmic and to possess three dileucine motifs that required for human Oca2 function. These motifs are essential for the targeting and localisation of Oca2 to the melanosome membranes, a function conferred by interaction with members of the clathrin-associated heterotetrameric adaptor protein family, AP-1 and/or AP-3 [10].

### Oca2 possesses a GOLD-like domain

The AlphaFold2 model predicts that the first luminal 130 residue loop forms an eight stranded beta sandwich. Examination of experimental PDB structures of other members of the IT superfamily revealed that Oca2 is the only member of the superfamily to possess this beta sandwich structure. Screening the 130-residue sequence of the beta sandwich against the PDB using HHpred did not yield any significant hits. To identify proteins possessing structurally similar regions, the 130-residue beta sandwich region was extracted from the AlphaFold2 model of Oca2 and screened, using Dali, against the full PDB. The results (Supplementary Table S2) gave a top hit with a Z-score of 10.3 for the central domain of tripeptidyl-peptidase 2 (TPP2) which is involved in the oligomerisation of TPP2 [45]. The other top hits from the screen are for proteins possessing the Golgi Dynamics (GOLD) domain and have Z-scores above 9. Although this region shows no sequence similarity to known GOLD domains, the identification of homology through structural comparison is in line with previous studies that indicate that GOLD domains have low sequence identity even between family members [46].

The functions of the GOLD domain are largely unknown although there are indications that it is involved in the trafficking of proteins from the endoplasmic reticulum to other subcellular compartments [46]. Oca2 is known to become terminally glycosylated when it transits from a post-ER compartment to the Golgi [10]. Human Oca2 has three evolutionary conserved consensus N-glycosylation sites (Asn 214, 218, and 273) [10] within the putative GOLD domain [10] and it has been demonstrated that some ER-resident proteins undergo GOLD domain N-glycosylation which is important for their trafficking between the ER and Golgi [47]. Taken together, these results suggest that the region previously termed the N-terminal luminal loop in fact encodes a GOLD domain involved in the trafficking of Oca2 from the ER to the Golgi prior to localisation at melanosomal membranes.

### Mutations in the Oca2 putative pore region results in severe albinism

The pseudo inverse repeat – made up of a broken transmembrane helix, an amphipathic helix and a re-entrant loop packing against the broken transmembrane helix – forms a substrate-binding chamber possessing two flanking re-entrant loops, with Consurf [48] highlighting the N-terminal half of the re-entrant loops as highly conserved. Mutations in Oca2 disrupt melanin production within the melanosome. The defective melanin synthesis has been linked to altered melanosome luminal pH which is correlated with reduced chloride conductance across the melanosome membrane [8].

Positions of mutations that are characterised *in vitro* or *in vivo* were mapped onto the AlphaFold2 model in order to provide a structural context. Mutations at positions V443 and K614 are known to result in oculocutaneous albinism type II as well as inhibiting *in vitro* melanosome melanin content [8]. Mapping these mutations onto the Alphafold2 model reveals that V443 is present on the N-terminal re-entrant loop of the first repeat unit and has a critical impact on chloride conductance across the melanosome membrane [8]. The position of this functionally critical residue on a re-entrant loop is in accordance with other transporters where the re-entrant loop has a role in channel specificity [49]. K614 is present on the predicted cytoplasmic loop between the two inverse repeat units that would not contribute to any transport functionality of the protein; this is in agreement with the fact that *in vitro* studies show that K614 mutations have little effect on chloride conductance. K614 mutations present in albino patients who also possess additional Oca2 mutations [50], so that the K614 change may itself not be of critical phenotypic importance.

Furthermore, mapping the quintuple mutation from Bellono et al. (V443I, M446V, I473S, N476D, N489D) [8] shows that the mutated positions are all present on the N-terminal re-entrant loop/transmembrane helix structural motif of the first repeat. Again, these mutations result in inhibition of chloride conductance and are present in the putative pore region of the protein [8].

**Figure 5:**
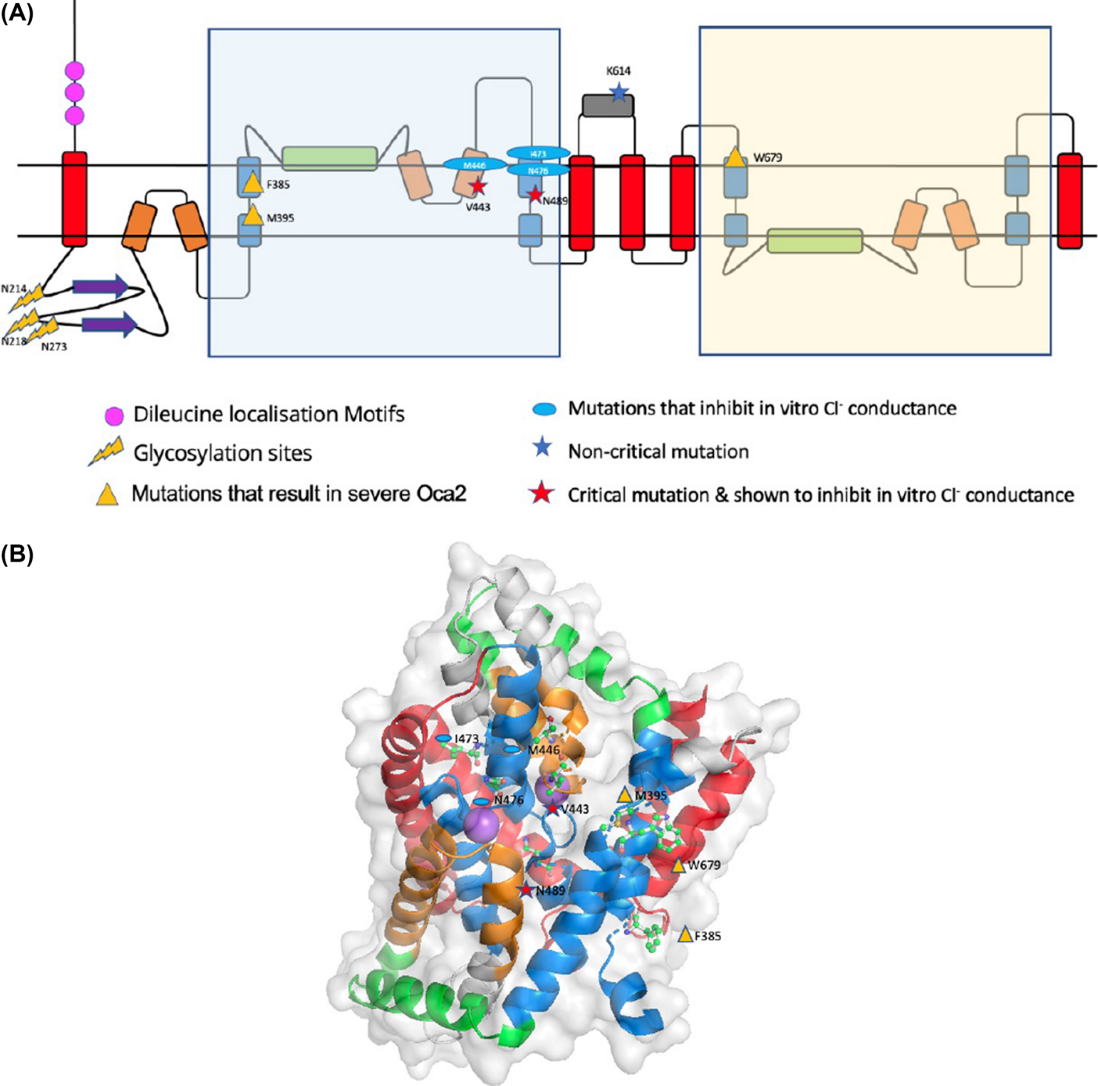
Mutation mapping of Oca2 (**A**) Oca2 topology with trafficking and example mutation sites mapped. (**B**) Example mutations mapped on to the AlphaFold2 Oca2 structure (ball and stick): residues are labelled and assigned the same symbols as in Figure 5A. The Na^+^ ions shown as purple spheres, positioned according to a superposition with 7jsk, marking the position of the putative ligand binding site.

Mapping of other known mutations that result in severe albinism (F385, M395, N489, N679) [50–52] show that these critical residues are present in either the N- or C-terminal transport domain region; F385 and M395 in the first transmembrane helix of the N-terminal transport domain region, N489 in the second transmembrane helix of the N-terminal transport domain region that packs with the re-entrant loop and W679 in the first transmembrane helix of the C-terminal transport domain region (Figure 5).

The mapping of known Oca2 mutations shows that those present in the inverted repeat that forms the putative pore are more likely to result in the severe oculocutanous albinism type 2 phenotype. Similarly, mutation of Oca2 in regions important for melanosome localisation also results in the severe phenotype of oculocutanous albinism type 2. However, in contrast, mutations localised relatively distant to the putative pore region do not result in the severe phenotypes of oculocutanous albinism type 2.

### Citrate docks at the putative binding site

Bellono et al speculate that Oca2 might be an accessory subunit of a Cl^−^ transporter or form a Cl^−^ channel or carrier protein itself [8]; this would be analogous to the bacterial homologue ArsB [30]. The pseudo inverted repeat topology that includes re-entrant loops facing each other in the membrane packed against transmembrane helices has been seen previously in other chloride transporters such as CLC transporters [49,53]. However, Oca2 docking of chloride was not successful; chloride did not dock at the putative binding site and was placed outside of the transport domain. Consequently, as Oca2 has strong HHpred hits with SLC13 transporters, docking of the SLC13 substrate was considered.

**Figure 6:**
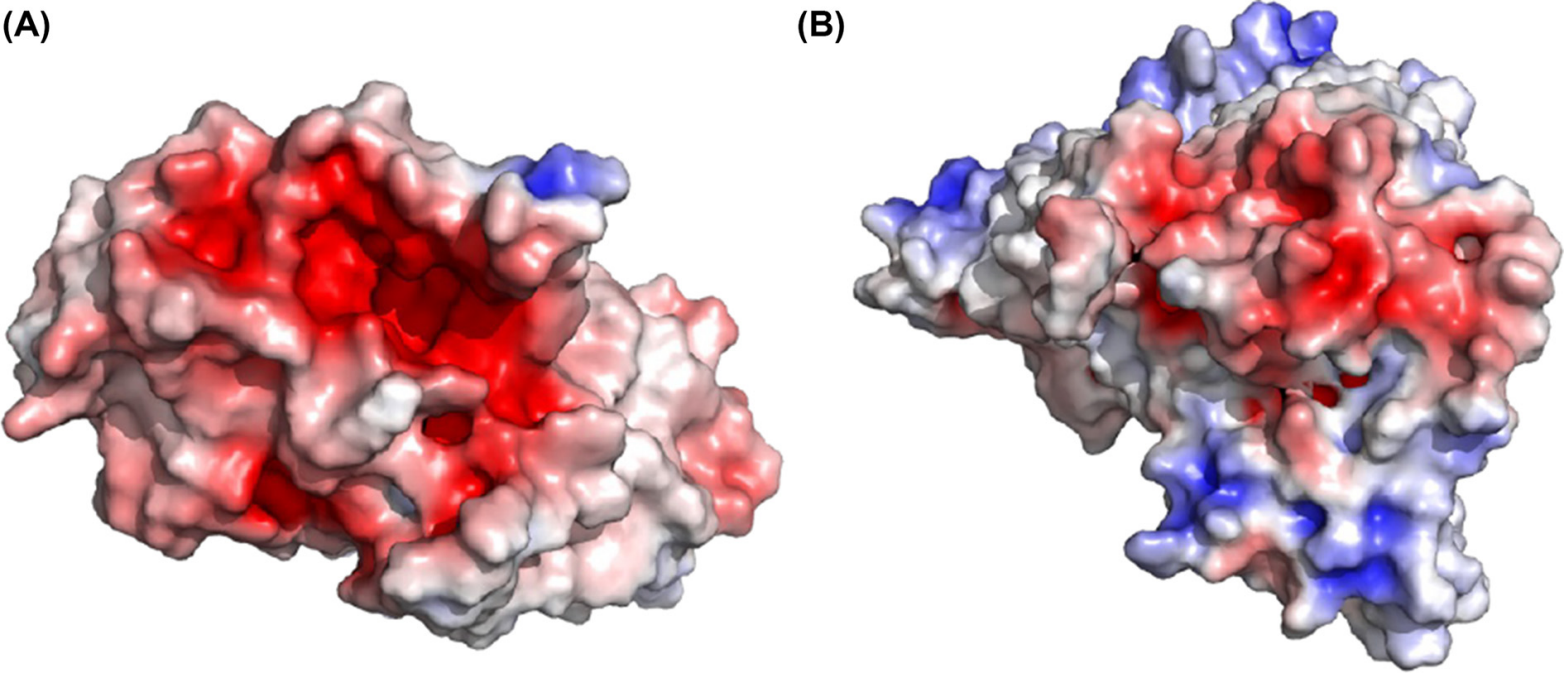
APBS electrostatics (adaptive Poisson–Boltzmann Solver) analysis revealing negatively charged regions (**A**) APBS electrostatic mapping surface view of Oca2 (transport domain only). (**B**) APBS electrostatic mapping surface view of NaCT. Spectrum: Red (negative) to blue (positive).

SLC13 transporters are members of the larger divalent-anion sodium symporter (DASS) family [54–57]. Most DASS transporters are sodium-coupled symporters that transport one substrate for each 2–4 sodium ions. However, some DASS members are antiporters [58]. Sequence analysis and examination of experimental models show that DASS symporters and antiporters share the same fold [59]. The DASS antiporters possess surrogate residues (K, R, or H) to compensate for the absence of sodium ions. Examination of Oca2 at the surrogate residue equivalent positions shows that the surrogate residues (K, R, or H) that are present in the experimental structure of the DASS antiporter 6wu1 (LaINDY) are not present in Oca2. Furthermore, the substrate binding residues where the side chain is involved in the binding of two sodium ions identified in the DASS symporter experimental structure of 7jsk (NaCT) (Asn141 and Asn465) are present at the equivalent positions in the Oca2 model (Asn442 and Asn741). Indeed, visualization of the electrostatic surface view of Oca2 highlights a negatively charged region corresponding to the putative Na^+^ binding site (Figure 6).

**Figure 7:**
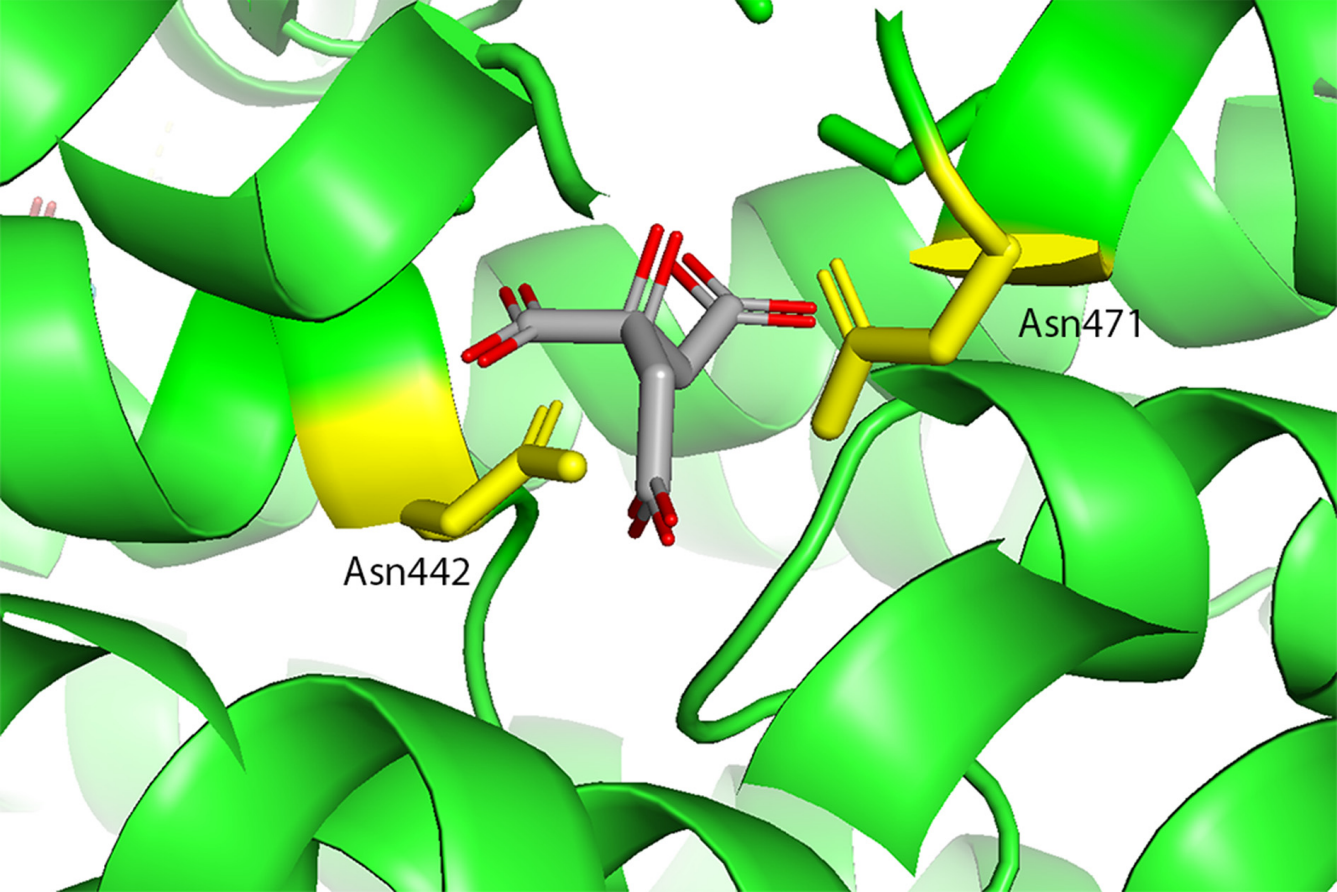
Webina docking of citrate to the Oca2 model Yellow sticks are used for the conserved Asn442/Asn741 residues; grey sticks are the citrate.

In the human NaCT experimental structure [35] a density which appears to be citrate is observed. There, in addition to Asn141 and Asn465, the authors propose additional substrate binding residues that form a substrate binding motif for citrate (Ser140-Asn141-Thr142 and Ser464-Asn465-Val466). The Oca2 model only possesses the Asn equivalents. Webina [23] was subsequently employed in an attempt to dock citrate at the equivalent position in Oca2 as seen in NaCT. The citrate does indeed dock on Oca2 at the equivalent position as observed in NaCT (Figure 7). Furthermore, docking of citrate to the AlphaFold2 model of NaCT results in docking again at the equivalent position with similar scores (Δ*G*: −6.3 kcal/mol and --5.1 kcal/mol for NaCT and Oca2, respectively). To confirm this, a rescoring exercise using Prodigy [25] was performed for both Oca2 and NaCT; again, both NaCT and Oca2 obtained similar scores of Δ*G:* −5.4 kcal/mol and --5.5 kcal/mol, respectively, supporting the Webina binding affinity energy scores. Attempting to dock citrate on to ArsB, however, does not result in docking of citrate at the putative binding site.

These results suggest that citrate is a plausible substrate for Oca2, but this does not immediately align with the experimental observation that Oca2 is involved with chloride conductance across the melanosome membrane. Dicarboxylates are known to have a role in metabolic signaling. It is plausible that the movement of citrate (or another dicarboxylate) in and out of the melanosome could modulate chloride conductance across the melanosomal membrane. Indeed, it has been shown that citrate inhibits melanin synthesis via the GSK3*β*/*β*-catenin signalling pathway which involves the regulation of tyrosinase transcription factors [60]; citrate may be involved in the regulation of melanin synthesis at other key points in synthesis pathway.

### AlphaFold2 multimeric modelling protocol in combination with traditional homology modelling models Oca2 in alternative conformations

**Figure 8:**
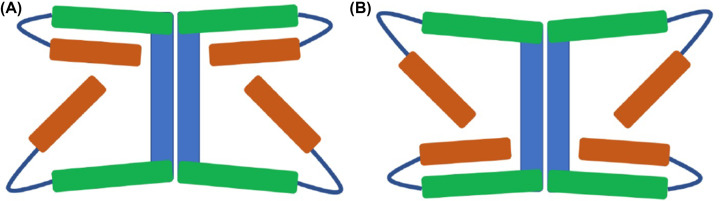
Schematic view of key Oca2 structural changes resulting from conformational movements (**A**) Inward-facing state. (**B**) Outward-facing state. Blue: scaffold domain; Green: amphipathic helix; Orange: N-terminal of re-entrant loop. Between the inward and outward states, the angles at amphipathic (green)/re-entrant loop (only N-terminal side showing - orange) hinges (dark blue) change by around 30° resulting in the movement of the transport domain (not shown) relative to the scaffold domain.

Given that DASS proteins operate via an elevator-type transport mechanism [36,61], and given the obvious homology that Oca2 shares with DASS transporters, it can be confidently predicted that the Oca2 transport mechanism is also of the elevator type. The elevator-type transport mechanism involves the sliding of the transport domain through the bilayer as a rigid body while the scaffold domain remains fixed in order to achieve the transitions between the outward- and inward-facing states [62]. During the transport cycle it has been demonstrated that DASS symporters cotransport by binding sodium first and then their substrate, with the reverse occurring during the release [39–41, 63, 64]. During the course of transition between the outward and inward-facing states, the angle between the amphipathic helices and the re-entrant loops changes by approximately 30° allowing the movement of the transport domain (Figure 8). The presence of flexible hinge loops between the amphipathic helices and the re-entrant loops facilitates transport domain movement [35].

Examination of the AlphaFold2 model showed that it was in the inward-facing state where the angle between the N-terminal half of the re-entrant loop and the amphipathic helix is approximately 30° for the N-terminal side and approximately 55° for the C-terminal side. As observed for the DASS transporters VcINDY and LaINDY; the N-terminal angle increases by around 30° and the C-terminal angle decreases by around 30° when the transporter switches to the outward-facing state [35].

Attempts were made to model Oca2 in an outward-facing conformation; building Oca2 locally using ColabFold resulted in 5 models all in the inward-facing conformation. Previously, feeding ColabFold shallow MSAs has proved effective for sampling the conformational space of transporters [42]. However, this strategy also produced models solely in the inward-facing conformational state. Further attempts were made to model Oca2 in an outward-facing conformation by a combination of utilizing the VcINDY outward-facing structure as a template for AlphaFold2 [20] and by providing AlphaFold2 with reduced depth MSAs. However, again, AlphaFold2 was only able to generate the inward-facing conformation. The inability to obtain the outward-facing conformation may relate to the proportions of different states in the PDB. The AlphaFold2 prediction neural networks were trained on all structures deposited in the PDB on or before April 30, 2018 [13], and many DASS homologues in the inward-facing state were deposited before this date with the few outward-facing examples being deposited in 2021 [35].

The failure to generate Oca2 in an alternative conformation forced us to develop a novel strategy. A homology model of Oca2 was built using the VcINDY outward-facing structure (6wtw) as a template for Modeller [21]. The output structure had a Dali alignment *Z*-score with 6wtw of 54.8: however, the Oca2 structural features that are not present in VcINDY such as the putative GOLD domain were obviously not modelled. To rectify this, the Modeller model was then used in ColabFold in conjunction with various custom MSAs of varying depths to build a series of Oca2 structures (Table 4).

**Table 4:** Structural characterisation of Oca2 models and template

		Dali *Z*-score vs 6wtw	Angle (°) between amphipathic helix and N-terminal side of re-entrant loop (N-terminal)	Angle (°) between amphipathic helix and N-terminal side of re-entrant loop (C-terminal)	Mean pLDDT
Inward facing model	Inward-facing AlphaFold2 model of Oca2	7.8	32	52	73.3
Outward-facing templates	Outward-facing template (6wtw)	–	64	15	–
	Modeller homology model (based on 6wtw) of outward-facing conformation	54.8	64	15	–
ColabFold models at various MSA depths	200 sequences	25.0	37	36	82.9
	60 sequences	30.9	51	28	73.0
	30 sequences	30.0	57	20	72.8
	15 sequences	32.9	59	22	70.0
	10 sequences	28.3	59	24	66.4
	5 sequences	14.0	43	23	57.7

**Figure 9:**
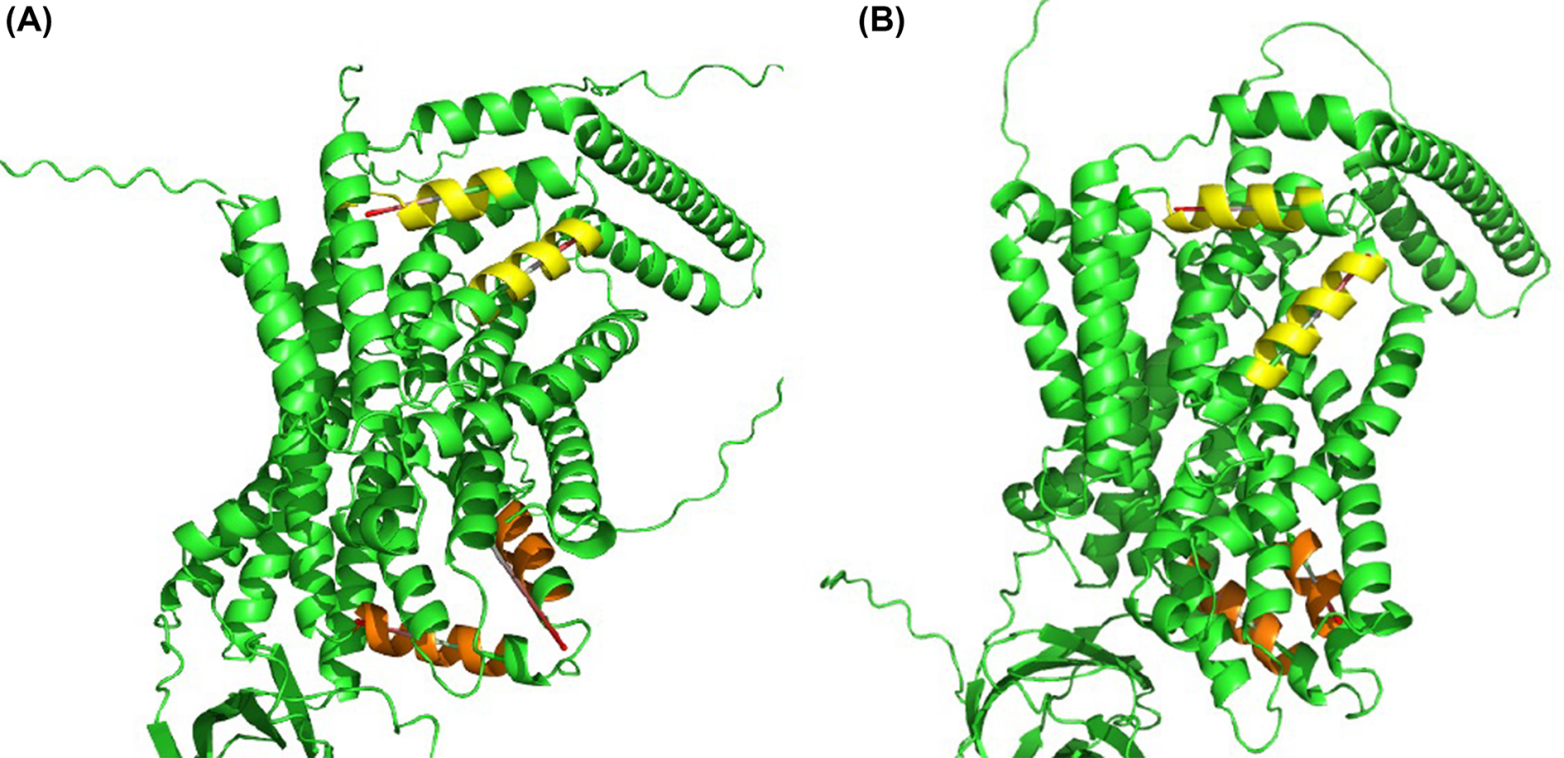
Monomeric Oca2 models in alternate conformational states (**A**) Inward-facing state. (**B**) Outward-facing state. Yellow: N-terminal amphipathic/re-entrant hinge; Orange: C-terminal amphipathic/re-entrant hinge.

The output models displayed the characteristic inverse repeat architectures of the scaffold and transport domains as seen in DASS transporters as well as the putative GOLD domain as predicted in the earlier modelling of the inward-facing state of Oca2. Reducing the MSA depth did negatively influence the quality scores of the output models but at the same time improved the Dali alignment *Z-*score with respect to the outward-facing DASS transporter 6wtw thereby indicating more outward-facing structures. Additionally, the measurement of the N-terminal amphipathic/re-entrant hinge angle and C-terminal amphipathic/re-entrant hinge angle showed the characteristic angle combinations of the outward-facing state; N-terminal angle being larger than the C-terminal angle (Figure 9).

### AlphaFold2 can construct a plausible Oca2 homodimer in both conformations when used in combination with traditional homology modelling

**Figure 10:**
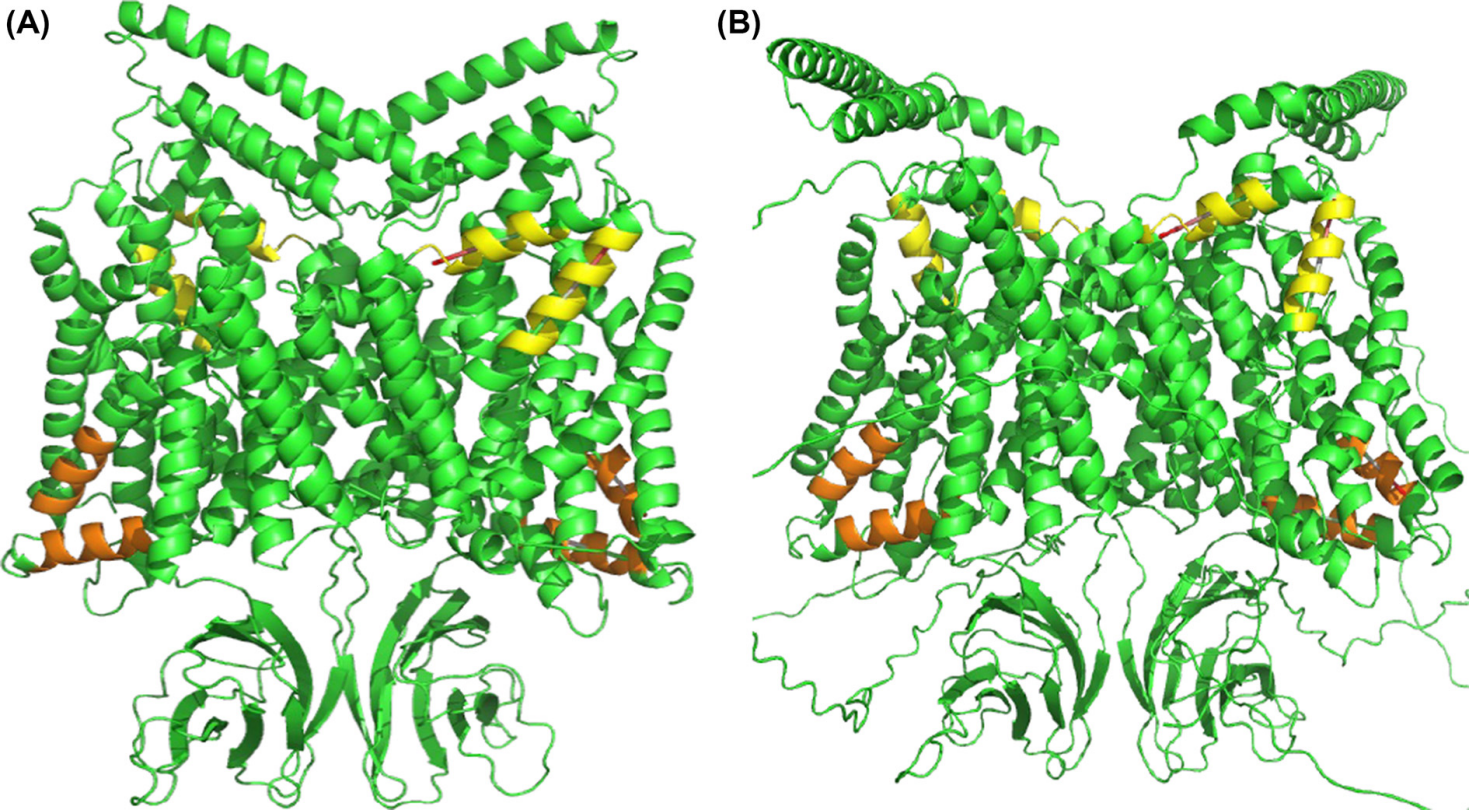
Homodimeric Oca2 models in alternate conformational states (**A**) Inward. (**B**) Outward. Yellow: N-terminal amphipathic/re-entrant hinge; Orange: C-terminal amphipathic/re-entrant hinge.

The close homologues of Oca2 have experimental structures that form homodimers [35,43], therefore, attempts were made to model Oca2 as a homodimer. First, ColabFold was employed to build a homodimer without using a template and utilizing the full MSA. This produced five models each of which were in the inward-facing conformation (Figure 10). Next the Modeller template and reduced MSA (15 sequences) were used to build a homodimer; this produced five models in the outward-facing conformation (Figure 10).

**Figure 11:**
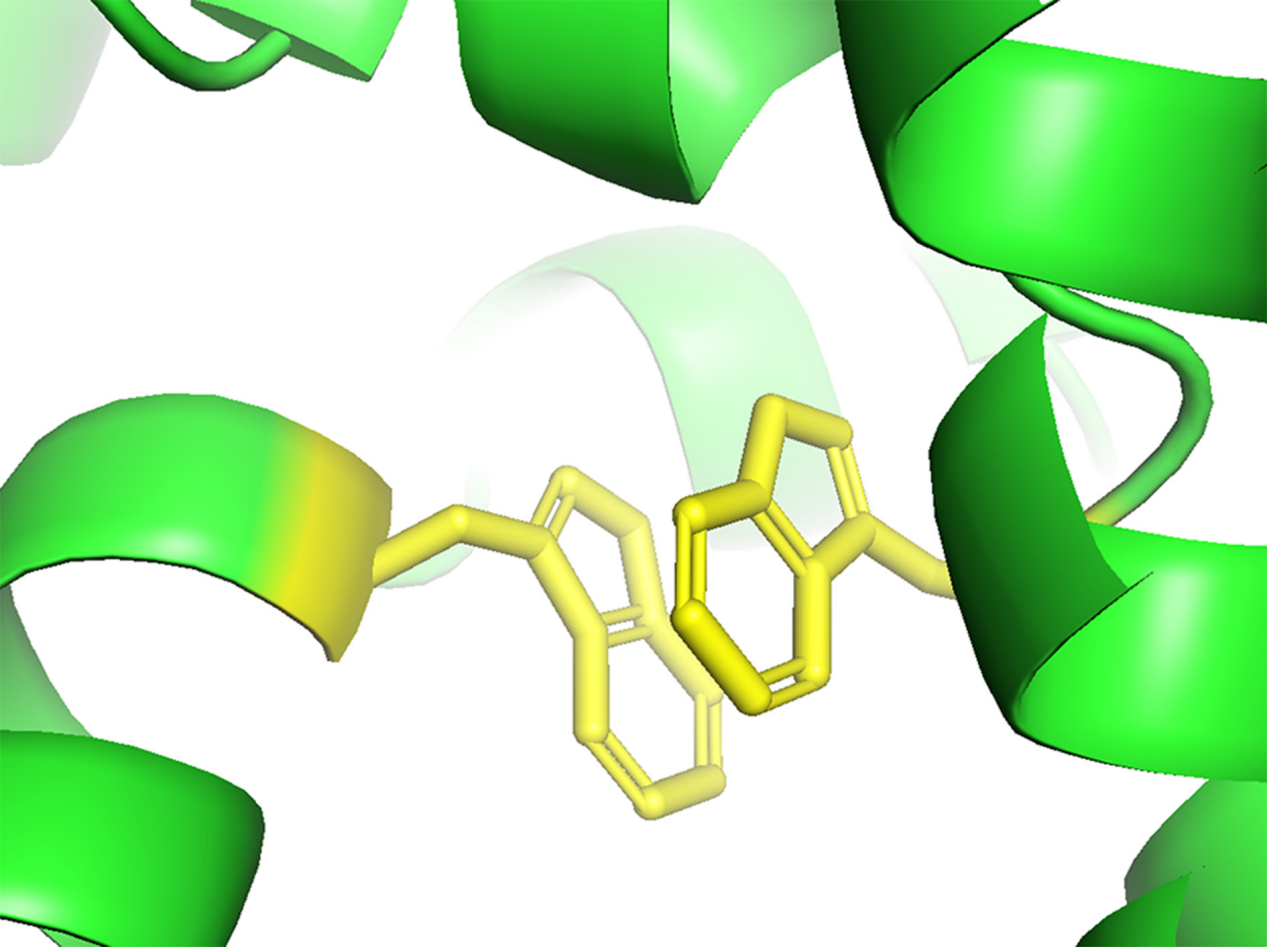
Homodimer interface Pi–Pi interaction Potential Pi-Pi interaction between Trp679 and Trp679’ (yellow) stabilising the Oca2 homodimer interface.

Pisa (Protein Interfaces, Surfaces and Assemblies) [44] was employed to determine the area of the interface as 55111 Å^2^. This interface area is much larger than that of NaCT; 21035 Å^2^. The presence of the putative GOLD domains in Oca2 contributes to this dimerization surface area resulting in this unusually large interface. To our knowledge, dimerised GOLD domains have not been reported previously. Further analysis of the homodimer interfaces of Oca2 revealed the presence of an interaction between Trp679 of both monomers. This could contribute to the stabilisation of the interface formed from the two scaffold domains resulting in a stable rigid structure in the membrane (Figure 11); this same interaction can be seen in the dimer interface of NaCT where Pi–Pi interaction between Trp408 and Trp408′ from the neighbouring protomers stabilises the two scaffold domains together into a rigid framework [35].

## Conclusion

Deep learning modelling methods were employed here to model Oca2. These methods have previously been shown to accurately model transmembrane proteins. DMPfold, for example, has been shown to be able to model transmembrane proteins with a TM-score of at least 0.5 to the native structure and obtain a mean TM-score of 0.74 [11]. The high accuracy of AlphaFold2 transmembrane protein modelling has been confirmed, for example, by exploring the construction of structures from the ATP-Binding Cassette (ABC) protein superfamily [14]. Although specific membrane protein benchmarking with trRosetta has not been reported, it has been shown that the trRosetta and the AlphaFold2 approaches generally agree with 50% of AlphaFold2 structures having a root-mean-square deviation (rmsd) Cα distance of less than 2 Å from the equivalent trRosetta model [65].

The AlphaFold2 model of Oca2 has provided the opportunity to revise the current consensus view of its topology. The Alphafold2 model predicts that Oca2 shows structural similarities to SLC13 proteins as well as strongly suggesting that it shares the DASS family topology and possesses a GOLD-like domain. The DASS family contains both symporters and antiporters; Oca2 possesses symporter features. Although the molecular specificity of Oca2 remains unclear, Oca2 possesses key citrate-binding residues as seen in NaCT and citrate docks to Oca2 at the putative binding site as observed in NaCT. When clinically relevant and *in vitro* tested mutations are mapped on to the model, it is seen that they cluster on the pore region of the transport domain of the structure. Analogously, clinically relevant mutations of NaCT that result in the insufficient transport of citrate, and thereby interrupt brain energy metabolism resulting in epileptic encephalopathy, are located on the re-entrant loops and broken helices of the pore region in NaCT [66]. Finally, AlphaFold2 modelling of Oca2 has demonstrated that, like DAS transporters, Oca2 can exist in two plausible conformations: inward- and outward-facing, supporting an elevator-type transport mechanism.

## Supplementary Material

Supplementary Tables S1-S2

## Data Availability

Figshare: Inward and outward-facing dimer models: https://doi.org/10.6084/m9.figshare.21786527.v1

## Open Access

Open access for this article was enabled by the participation of University of Liverpool in an all-inclusive *Read & Publish* agreement with Portland Press and the Biochemical Society under a transformative agreement with JISC.

## Abbreviations

ABC: ATP-Binding Cassette
DASS: divalent-anion sodium symporter
NACT: sodium-dependent citrate transporter
RPE: retinal pigment epithelium
SLC: solute carrier
