# Supplementary material for: Structural insights into pink-eyed dilution protein (Oca2)
Source: Biosci Rep. 2023 Jul 25;43(7):BSR20230060. doi: 10.1042/BSR20230060 (PMC10372470; doi:10.1042/BSR20230060)
Supplement: Supplementary Tables S1-S2 [file BSR-2023-0060_supp.pdf]

Supplementary Table1: DaliresultsforstructuralscreenofOca2againstPDB

PDBcode	Z-Score	Name
7jsj-A	39.1	SoluteCarrierFamily13Member 5;
6ol1-D	37.7	SoluteCarrierFamily13Member 5;
5ul7-A	37.5	Transporter,NadcFamily;
6okz-C	36.3	Transporter,NadcFamily;
5uld-D	36.0	Transporter,NadcFamily;
6ol0-D	36.0	Transporter,NadcFamily;
6wtx-D	35.3	DassFamilySodium-CoupledAnionSymporter;

Supplementary Table2: Daliresultsforstructuralscreenofbetasandwich (residues 196-331)regionagainstPDB

PDBcode	Z-Score	Name
3lxu-X	10.3	Tripeptidyl-peptidase2;
6q69-C	9.6	Peripherialbenzodiazepinereceptorassociatedpr
5azw-B	9.4	Transmembraneemp24domain-containingprotein2;
4uyb-A	9.3	Sec14-likeprotein3;
5lz3-A	9.2	Golgiresidentproteingcp60
1o6u-C	9.2	Sec14-likeprotein2;
1olm-A	9.1	Sec14-likeprotein2;
